# Optogenetic activation of neocortical neurons *in vivo* with a sapphire-based micro-scale LED probe

**DOI:** 10.3389/fncir.2015.00025

**Published:** 2015-05-29

**Authors:** Niall McAlinden, Erdan Gu, Martin D. Dawson, Shuzo Sakata, Keith Mathieson

**Affiliations:** ^1^Institute of Photonics, Department of Physics, University of StrathclydeGlasgow, UK; ^2^Strathclyde Institute of Pharmacy and Biomedical Sciences, University of StrathclydeGlasgow, UK; ^3^Centre for Neuroscience, University of StrathclydeGlasgow, UK

**Keywords:** optogenetics, neurotechnology, cortical layers, neural circuit, μLEDs

## Abstract

Optogenetics has proven to be a revolutionary technology in neuroscience and has advanced continuously over the past decade. However, optical stimulation technologies for *in vivo* need to be developed to match the advances in genetics and biochemistry that have driven this field. In particular, conventional approaches for *in vivo* optical illumination have a limitation on the achievable spatio-temporal resolution. Here we utilize a sapphire-based microscale gallium nitride light-emitting diode (μLED) probe to activate neocortical neurons *in vivo*. The probes were designed to contain independently controllable multiple μLEDs, emitting at 450 nm wavelength with an irradiance of up to 2 W/mm^2^. Monte-Carlo stimulations predicted that optical stimulation using a μLED can modulate neural activity within a localized region. To validate this prediction, we tested this probe in the mouse neocortex that expressed channelrhodopsin-2 (ChR2) and compared the results with optical stimulation through a fiber at the cortical surface. We confirmed that both approaches reliably induced action potentials in cortical neurons and that the μLED probe evoked strong responses in deep neurons. Due to the possibility to integrate many optical stimulation sites onto a single shank, the μLED probe is thus a promising approach to control neurons locally *in vivo*.

## Introduction

Since the early 2000s (Boyden et al., 2005), optogenetics has become one of the standard experimental techniques in neuroscience (Yizhar et al., 2011; Häusser, 2014). Optogenetic approaches have been successfully applied to modulate neural activity in a cell-type-specific manner (Adamantidis et al., 2007; Huber et al., 2008; Cardin et al., 2009; Packer et al., 2013), allowing scientists to functionally dissect diverse types of neurons even in behaving animals. However, it is still difficult to control the neural activity of a particular cell-type at high spatial resolution *in vivo* (Rickgauer and Tank, 2009; Rickgauer et al., 2014). For example, neocortical circuits have anatomically prominent six-layered structures, where certain cell-types are distributed across these cortical layers. These neurons can have layer-specific functions, but it is challenging to target them for *in vivo* stimulation using currently available optical stimulation technology and without spatially restricted gene expression.

Recently, novel approaches have been proposed to deliver light into the brain: such as monolithically integrated dielectric waveguide and recording electrodes (Wu et al., 2013), a coaxial optrode (Wang et al., 2012; Ozden et al., 2013), a silicon probe with multiple diode fibers (Stark et al., 2012), fluorescence microendoscopy (Hayashi et al., 2012), multipoint-emitting optic fibers (Pisanello et al., 2014) and a three-dimensional multiwaveguide probe (Zorzos et al., 2012). With waveguide and fiber optic approaches, scaling up the number of optical stimulation sites without increasing the probe dimensions to an extent where substantial neural damage occurs during insertion will always be a problem. Two-photon excitation has also been shown to excite neurons (Rickgauer and Tank, 2009; Rickgauer et al., 2014). However, in each of these cases the light sources are located external to the neural tissue and require complex optical components to scale the number of illumination sites. This leads to an expensive and technically challenging system. An alternative approach is to integrate micron-scale light sources on a probe itself and micro light-emitting diodes (μLEDs) are a promising solution in this regard. Recently we (McAlinden et al., 2013) and other groups (Kim et al., 2013; Moser, 2015) have independently proposed μLED technology for *in vivo* optogenetic experiments.

The μLED technology is based on gallium nitride (GaN) LEDs with novel micro-pixellated configurations, which can emit high intensity light to be delivered at high spatiotemporal resolution (Zhang et al., 2008). This technology has been successfully applied for optogenetic stimulations of brain slices *in vitro* (Grossman et al., 2010). Using a novel pick-and-place technique, Kim et al. (2013) recently integrated inorganic μLEDs on a polymer substrate with a multifunction probe. This is the first realization of cellular-scale optoelectronic technology in behaving animals. However, progress needs to be made in developing μLED probes that can be *individually* controlled to allow spatiotemporal patterned activation of optogenetic actuators at high spatial resolution.

In previous work we developed a custom GaN-based μLED probe and characterized the electrical, optical and thermal properties (McAlinden et al., 2013). This prototype has up to five μLEDs on each probe fabricated on a thinned sapphire substrate, which is strain matched to the GaN layers and allows high-brightness μLEDs. However, sapphire is a challenging material to process, resulting in a minimum thickness of 100 μm. Each μLED emits at a peak wavelength of 450 nm with an irradiance of up to 2 W/mm^2^ (at the surface of μLED) and is independently addressable through electrical contacts attached to a current source. We adopted a broad range of operation regimes in stimulation mode, confirming that local increases in brain tissue temperature can be kept minimum (<0.5°C), while operating at intensities and pulse durations needed for *in vivo* optogenetic experiments. Here we report on the *in vivo* performance of this prototype device.

## Materials and Methods

### Probe Design, Production and Characterization

**Figure 1:**
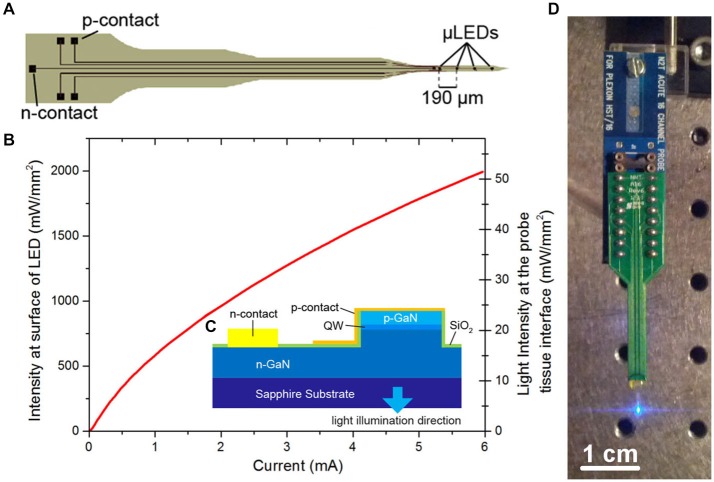
**μLED probe. (A)** Schematic of μLED probe, showing four μLEDs at the tip, the single *n*-contact and bonding pads linked with tracks to the *p*-contacts for each μLED. **(B)** Light output characteristics of one of the μLEDs on the neural probe at the surface of the μLED and at the tissue/probe interface. **(C)** Schematic cross section of the Sapphire-based probe (QW, quantum well). **(D)** Final device mounted onto a printed circuit board (PCB) and connected to a programmable current source.

The μLED probe was fabricated as described in McAlinden et al. (2013) from a commercial GaN LED wafer (SuperNovaOpto) with a sapphire substrate thinned post-growth to 100 μm. This material was chosen as it allows for the production of efficient, high brightness μLEDs. The probe (Figure 1) is designed to have a length of 5 mm. The tip is 1 mm long and 150 μm wide and has a tapered design to minimize damage during insertion into tissue. Each tip contained four μLEDs with a pitch of 190 μm and a diameter of 30 μm. The other end of the probe contains bond pads allowing each μLED to be individually addressed. Laser dicing was used to etch trenches through the sapphire and release individual probes. Following dicing each probe was glued to a printed circuit board (PCB) and the device was wire-bonded. The wire bonds were protected using a UV curable polymer (Norland) and the device was electrically isolated by conformal deposition of a 3 μm thick transparent parylene layer. A full list of the materials used and their sources is shown in Table 1. The fabricated probes were characterized spectrally, electrically and optically (Figure 1B) where the power was measured with a calibrated photodiode placed directly above the sapphire surface of the μLED under test. The irradiance at the emitting surface of the μLED was calculated from this externally measured power by accurately measuring the geometry of the system and using simple ray tracing.

**Table 1:** **List of material sources**.

Description	Supplier	Website
GaN wafers: 3″ GaN-on-Sapphire, λ = 450 nm	SuperNova Optoelectronics Corp.	http://www.supernovaopto.com
PCB: bare PCB, model A16	Neuronexus Technologies, Inc.	http://neuronexus.com
Norland UV curable adhesive, NOA 68	Norland Products Inc.	https://www.norlandprod.com
Parylene C, conformal coating, 1 μm thick	Para Tech Coating Ltd	http://www.paratechcoating.co.uk

### Monte Carlo Simulation

The μLED probe devices presented here have a complex emission profile. The μLED will emit light in a Lambertion profile, however the light must pass through 100 μm of sapphire before reaching the neural interface. Light can also become trapped in this sapphire layer due to total internal reflection. To estimate the intensity of light at various distances from a μLED, a Monte Carlo simulation was performed using custom-written software. The program stimulated >10^6^ photons starting from random positions on the 30 μm diameter LED surface, and moving in a random direction (weighted by a Lambertion distribution). Common to Monte Carlo particle transport, the photon is moved in discrete steps between interaction sites with the step size determined using the inverse distribution method and the Beer–Lambert law (Wang et al., 1995). At each interaction site a proportion of the photon weight is absorbed and the photon is scattered, changing its direction of travel. When the photon weight drops below a threshold value or the photon moves sufficiently far outside of the modeled area it is terminated and a new photon is launched. The custom software also accounts for total internal reflection in the 100 μm thick sapphire layer and refraction between the sapphire and brain tissue. A further modified simulation was used to study the light output from an optical fiber on the comparison optrode (see below). The optical fiber was 105 μm in diameter with a numerical aperture of 0.22. Each of the photons was initiated from a random position at the aperture of the fiber weighted by a Gaussian distribution. Particle trajectories were random within the bounds set by the numerical aperture. As in the experimental case, the simulated tip of the fiber was outside the brain and as such the light had to pass through 300 μm of saline before reaching the brain tissue. The optical constants of brain tissue used were, scattering anisotropy *g* = 0.88, scattering coefficient μ_s_ = 11.7 mm^−1^, absorption coefficient μ_a_ = 0.07 mm^−1^ and refractive index *n* = 1.36 (Yaroslavsky et al., 2002).

### Animals

All animal experiments were performed in accordance with the UK Animals (Scientific Procedures) Act of 1986 Home Office regulations and approved by the Home Office and University’s Ethical Committee (PPL 60/4217). Emx1-IRES-Cre (Jax#005628; Gorski et al., 2002) and Ai32 (Jax#012569; Madisen et al., 2012) were crossed to express ChR2 (H134R) in the neocortex. Four Emx1-IRES-Cre; Ai32 mice (male, 20–30 week old, 33–41 g) were used.

### Surgery

After animals were anesthetized with 1.5 g/kg urethane, the animal was placed in a stereotaxic frame (Narishige) and body temperature was retained at 37°C using a feedback temperature controller (40-90-8C, FHC). After incision, the bone above the left sensorimotor cortices (0–1 mm posterior from the bregma, 0–1 mm lateral from the midline) was removed and the cavity was filled with warm saline during the entire recording session. The μLED probe was slowly inserted into the cortex with 10° angle to the normal and penetrated 1.5 mm. A 32 channel silicon-based optrode (A1 × 32–10 mm-50-177-A32OA, NeuroNexus Technologies) was inserted slowly (~2 μm/sec) and penetrated up to 1.25 mm with a motorized manipulator (DMA-1511, Narishige). The distance between the μLED probe and optrode was between 200–500 μm at the cortical surface. Several penetrations were made for each animal. For histological verification of tracks, the rear of the probe was painted with DiI (D-282, ~10% in ethanol, Molecular Probe; Sakata and Harris, 2009).

### Electrophysiology and Optical Stimulation

For electrophysiological recording, broadband signals (0.07 Hz 8 kHz) were amplified (×1000) (HST/32V-G20 and PBX3, Plexon) relative to a cerebellar bone screw and were digitized at 20 kHz (PXI, National Instruments). Once spiking activity was detected, optical pulses (50 ms pulse width at 2 Hz repetition rate) were delivered from either the optical fiber or μLED to assess whether neurons could be activated by optical stimulation, after which recording sessions were initiated. Each recording session typically consisted of a non-stimulation period (up to 2 min), optrode and μLED stimulation periods (up to 3 min) and another non-stimulation period (up to 2 min). The μLED was driven by a current source (Yokogawa, Source Measure Unit GS610) from 0.1 mA up to 6 mA (Corresponding to intensities at the probe/neural tissue interface from 0.5 mW/mm^2^ up to 52 mW/mm^2^). In the experiment in Figure 3, the μLED was supplied with 4 mA (40 mW/mm^2^) current pulses. The light source of the optrode was a commercial GaN LED (450 nm, PlexBright, Plexon) with 58.9 mW/mm^2^ output at tip of the probe. This light level was used as standard for all cortical experiments as it allow for stimulation along the full length of the optrode.

### Histology

After the experiments, animals were perfused transcardinally with physiological saline followed by 4% paraformaldehyde/0.1 M phosphate buffer, pH 7.4. After an overnight postfixation in the same fixative, brains were immersed into 30% sucrose/phosphate buffer saline and cut into 100 μm coronal sections with a sliding microtome (SM2010R, Leica), and the sections were mounted on gelatin-coated slides and cover-slipped with a mounting medium (Vectashield, Vector Labs). Sections were observed in an epifluorescent upright microscope (Eclipse E600, Nikon) to verify probe tracks.

### Data Analysis

All spike detection and sorting took place offline. For this process, freely available software (KlustaSuite)^1^ was used. Spike train analysis was performed with Matlab (Mathworks). A two-sample *t*-test was performed to assess the statistical significance of the optically evoked responses. A *p*-value of less than 0.05 was considered significant.

### Publicly Available Data Set

The raw data recorded for this article can be found at http://dx.doi.org/10.15129/53ec9764-79b1-4746-b5bc-f45088b5a774.

## Results

### Characteristics of the μLED Probe

**Figure 2:**
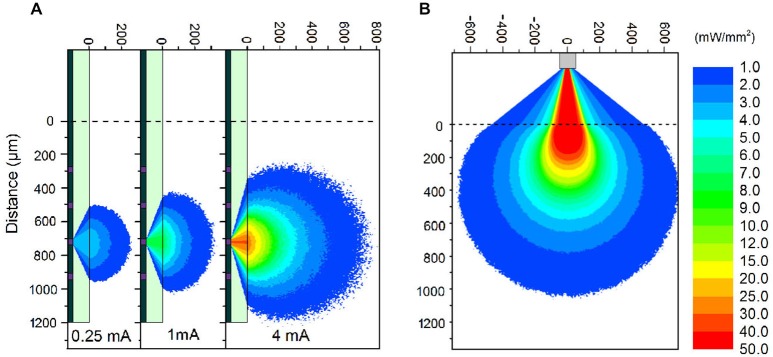
**Monte Carlo simulations. (A)** Simulations of light propagation from a μLED with a diameter of 30 μm through brain tissue. The simulation is repeated for 3 different input currents of 0.25 mA, 1 mA, and 4 mA, respectively. **(B)** Simulation of light propagation from the tip of a 105 μm diameter optic fiber through 300 μm of saline and then through brain tissue. The output power at the tip of the fiber was 1.1 mW. **Note**: The light propagation through the sapphire **(A)** and saline **(B)** is only representative. The true propagation involves back-reflections and strong interference patterns, which have been accounted for in the model, but detract from the clarity of the figure without contributing to the significance of the result.

The fabricated μLED probe demonstrated high light output, as shown in Figure 1B, with an irradiance at the μLED surface of 2 W/mm^2^ possible at 6 mA, the light will propagate through the sapphire substrate giving a maximum intensity of 52 mW/mm^2^ at the tissue/probe interface. The μLED probe has an emission spectrum that peaks at a wavelength of 450 nm and a full width at half maximum of 20 nm. A typical spectrum for this device can be seen in McAlinden et al. (2013). The input current can be modulated down to 100 μA dictated by the turn on voltage of the diode and giving an irradiance of 100 mW/mm^2^ at the μLED surface, which corresponds to 0.5 mW/mm^2^ at the tissue/probe interface. Since the device is driven by a current source, a certain voltage drives a fixed current through the diode structure. This voltage remains stable whether the device is operated in air, or saline demonstrating that the device is well electrically isolated by the PECVD oxide and parylene insulating layers, suggesting that no additional current paths arise from *in vivo* operation. The device has a reflecting p-metal contact on each micro-pixel, meaning that the light is predominantly emitted through the optically transparent sapphire substrate (Figure 1C). Since light travels through the sapphire substrate (Figure 1C) and follows a Lambertian distribution (Griffin et al., 2005), the effective diameter of an illumination site at the sapphire surface is larger than the physical diameter of the μLED (30 μm). This corresponds to a reduction in the intensity of light emerging at the sapphire/tissue interface and we have quantified this effect using the Monte Carlo method. Figure 2 compares the light distribution from the μLED probe and the optrode (see Section Materials and Methods). The fabricated μLED probe can activate volumes very similar to the optrode device, but can do this in a depth independent manner. Figure 2A shows the light propagation from a 30 μm μLED through a sapphire layer and into the cortical tissue for 3 different μLED currents; 0.25 mA, 1 mA and 4 mA, respectively. Using a low μLED current allows for small-area local stimulation, while using a higher current can give broad area stimulation, up to 0.3 mm^3^ with an operating current of 4 mA. In each case only the deeper cortical layers can be illuminated whereas superficial layers remain below the threshold for ChR2 activation. For comparison, Figure 2B shows the stimulation area for the optical fiber. At the light output shown, there is sufficient light to illuminate to a depth of 1.0 mm. Neurons in the superficial cortical layers will be strongly activated. Local excitation is always difficult using this approach and illumination of only deep cortical layers would be impossible. Based on these simulations, it is expected that cortical neurons at different depths (up to ~1 mm deep) can be activated with the conventional illumination, whereas deep cortical neurons will be activated with the μLED inserted to a target depth.

### Optogenetic Neocortical Neural Activation *In Vivo*

**Figure 3:**
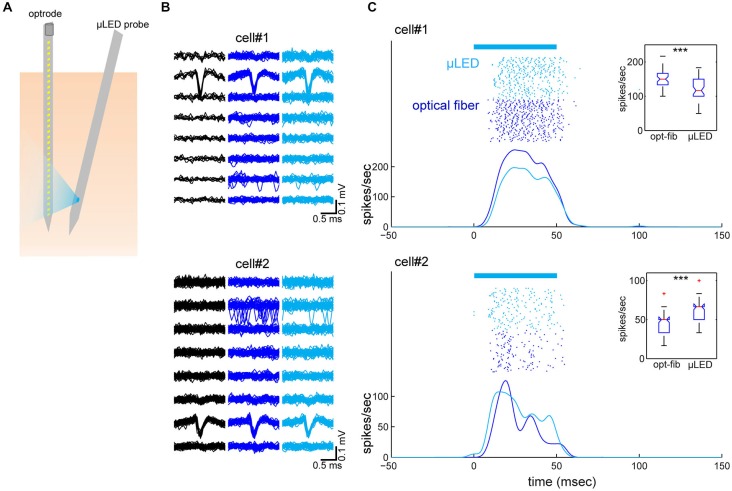
**Simultaneously recorded neocortical neurons and their optical responses. (A)** Schematic of the geometry of probe insertion into the neocortex. **(B)** Depth profiles of average spike waveforms of isolated single units. Signals from the bottom 8 channels are shown. *Black*, spontaneous activity. *Blue*, optical fiber stimulation. *Light Blue*, μLED stimulation. **(C)** Raster plots and peri-stimulus time histograms (PSTHs) for optically evoked responses. Fifty optical stimulation pulses, each 50 ms in duration were applied at a 2 Hz repetition rate, for both μLED (4 mA) (light blue) and fiber (58.9 mW/mm^2^) (blue) activation. The bar on the top indicates the timing of optical stimulation. PSTHs were smoothed by a 3-ms Gaussian kernel. Insets are boxplots of optically evoked responses for each condition and each cell (0–60 ms window from stimulus onset). ***p < 0.001 (two-sample *t*-test).

To test our predictions, we performed *in vivo* experiments where both an optrode and the μLED probe were inserted into the neocortex next to each other (Figure 3A). We used urethane anesthetized Emx-1-IRES-Cre; Ai32 mice that express ChR2(H134R) in the entire cortex. Four experiments were conducted in total, of which one experiment showed good recordings from two cortical neurons simultaneously with optically evoked responses using both optical fiber and μLED stimulation (4 mA; Figures 3B,C). In this experiment, the distance between the two probes was 400 μm at the cortical surface and the optrode and μLED probe penetrated to a depth of 1.2 mm and 1.5 mm, respectively, with 10° angle between them. First we estimated the depth position of the neurons measuring the peak position of the spike waveforms recorded on the 32-channel electrode array (Figure 3B). This estimate showed that the neurons were separated by ~250 μm in depth. We did not observe any distortion of spike waveforms during optical stimulation. Secondly, we assessed the optically evoked responses shown in Figure 3C. Although these two neurons spontaneously fired at 0.04 Hz and 0.1 Hz, respectively, as we expected, both deep cortical neurons were strongly activated by both types of optical stimulation.

**Figure 4:**
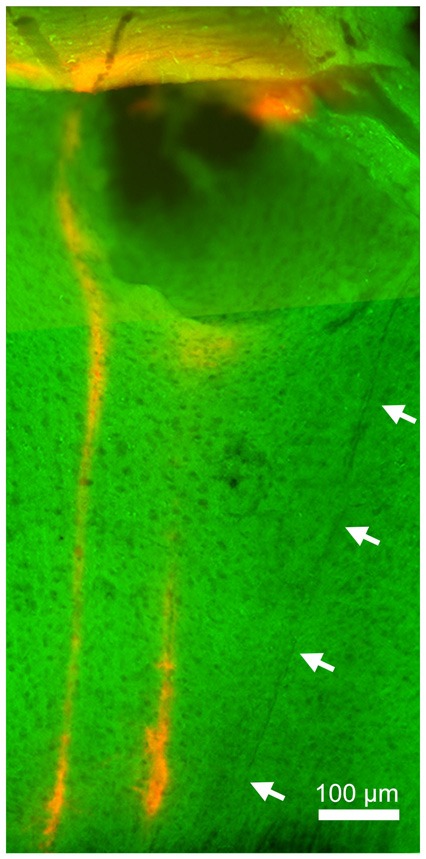
**Histology**. Green signals indicate enhanced yellow fluorescent protein (EYFP) expression in the sensorimotor cortex. Red signals indicate DiI signals, highlighting the position of the implanted optrode. The left optrode track shows the position of the optrode when robust, optically-driven responses were observed (see Figure 3). Arrows indicate the track of the μLED probe which is not as visible since it was not coated in DiI. Although the insertion of this 100 μm μLED probe caused damage in superficial layers, deep layers appeared relatively intact.

Finally, to verify the location of probes, we performed a histological analysis (Figure 4). Enhanced yellow fluorescent protein (EYFP) was expressed in the entire neocortical layers as expected (Gorski et al., 2002; Madisen et al., 2012). Although significant damage can be observed in superficial layers (due to the thickness of the μLED probe, 100 μm), the track of the μLED probe and the multiple tracks of the optrode can be clearly identified. The cortical tissue in the deep layers, where neuronal signals were recorded, appeared to be undamaged.

## Discussion

We have demonstrated the feasibility of sapphire-based μLED probes for *in vivo* optogenetic experiments, successfully activating neocortical neurons *in vivo*. Monte Carlo simulations show that the μLED probes are able to illuminate volumes equivalent to conventional techniques and that this illumination can be decreased so that local regions are activated. Additionally, the depth of light penetration no longer depends on light scattering and absorption, but on how far the probe is inserted into the brain.

In our experiments, μLED stimulation led to strong responses in deep cortical neurons. To ensure that the recorded waveforms were not optically induced artifacts, we recorded spontaneous activity and show that the spiking waveform matches the optically induced cases (both for the μLED and fiber, see Figure 3). This is further reinforced by the fact that the waveforms are recorded at different depths and are asynchronous in time. Though the spontaneous firing rates were low for the recorded neurons, it is not uncommon for cortical neurons to fire sparsely (Sakata and Harris, 2009, 2012). In addition, the current experimental protocol induced damage in superficial layers (Figure 4), which likely contributed to low spontaneous firing rates as losing synaptic inputs and damaging dendrites in superficial layers may affect firing rates in deeper layers. However, optical stimulations could elicit action potentials due to the strong expression of ChR2 in the somas. The exact neural mechanisms of the optogenetic activation are likely complex (i.e., direct and indirect effects) due to ChR2 expression patterns in the transgenic mouse used. However, our results do show that μLED stimulation can robustly elicit optogenetic neural activation in the neocortex and may induce a different pattern of neural population activity *in vivo* from that with a conventional approach.

### Comparisons with other Approaches and Future Perspective

Activation of cortical neurons with surface illumination is the most popular option to date (Huber et al., 2008; Cardin et al., 2009; Sachidhanandam et al., 2013; Zagha et al., 2013; Fu et al., 2014; Schneider et al., 2014; Zhang et al., 2014). However, given the fact that different cortical layers and cell-types contribute to different aspects of neural processing and coding (Sakata and Harris, 2009; Harris and Mrsic-Flogel, 2013; Huang, 2014; Kepecs and Fishell, 2014; Womelsdorf et al., 2014), such conventional approaches have a serious limitation in activating different cortical layers without specific gene expression strategies (Huang, 2014). This is especially true in the case of cell-types that are located across layers, such as GABAergic interneurons. The approach outlined here will offer a new option to activate a particular cell-type in a laminar-specific manner. Moreover, our approach is easily applicable to deeper brain structures, which have been targeted for deep brain stimulation.

Recently many new strategies for delivering light for optogenetic studies have been proposed (Hayashi et al., 2012; Stark et al., 2012; Wang et al., 2012; Zorzos et al., 2012; Ozden et al., 2013; Wu et al., 2013; Warden et al., 2014). These offer several advantages to the conventional approach including multiple excitation sites, activation at depth, incorporating electrodes onto the light delivery probe and *in vivo* imaging. Of particular interest are the proposed multipoint-emitting optic fibers (Pisanello et al., 2014) which have advantages in multipoint stimulations, multi-color illumination and fabrication costs and the multifunction probes made by Kim et al. (2013), which consist of four interconnected LEDs to give a large stimulation volume. The μLED probe discussed here shows the advantages of both of these technologies, including relative ease of manufacture, individually addressability of excitation sites, scalability to many excitation sites and potential miniaturization. A clear technology development strategy can also be seen to allow these probes be used for *in vivo* optogenetics studies in awake behaving animals. New technologies of this type are seen as key to future research in optogenetics (Deisseroth and Schnitzer, 2013; Warden et al., 2014).

Our current approach using GaN on sapphire material allows us to produce high brightness low leakage current μLEDs. However, there are 3 technical challenges that limit the use of this device. Firstly, sapphire is a challenging material to thin beyond 100 μm, making the probe more invasive that desired. Secondly, the μLEDs will generate heat at the surface placing upper limits on pulse duration and duty cycle (see McAlinden et al. (2013), where these parameters are explored). Thirdly, the spatial resolution is limited by the Lambertian emission profile of the μLED and the fact that light has to propagate through the sapphire substrate before reaching the neural interface. This may result in unwanted optogenetic activation, such as dendritic activation. To overcome these technical challenges we predict that moving to GaN grown on a silicon substrate (a material growth strategy being pursued commercially by the LED lighting community) will allow standard microfabrication techniques to be employed, resulting in thinner probes (below 50 μm) with increased functionality (e.g., multiple optical stimulation sites) and a higher yield. A silicon substrate will also enhance the thermal properties of the device due to the increased thermal conductivity. Spatial resolution can be optimized by producing top emission μLEDs, where light has only to transverse the encapsulating layers (typically a few microns in thickness) before interfacing with the neural tissue. Hybrid μLED probes with integrated recording electrodes can also be envisaged in the near future. Due to these possibilities, μLED probes are a promising approach to control neural activity locally at different depths *in vivo*.

## Conflict of Interest Statement

The authors declare that the research was conducted in the absence of any commercial or financial relationships that could be construed as a potential conflict of interest.

## Footnotes

^1^https://github.com/klusta-team
